# Social Vulnerability and Pregnancy Option Counseling in the Setting of Periviable Delivery

**DOI:** 10.3390/jcm14020466

**Published:** 2025-01-13

**Authors:** Gillian Piltch, Lizelle Comfort, David Krantz, Frank Jackson, Matthew J. Blitz, Burton Rochelson

**Affiliations:** 1 Northwell, New Hyde Park, NY 11042, USA; lcomfort@northwell.edu (L.C.); dkrantz@northwell.edu (D.K.); fjackson@une.edu (F.J.); mblitz@northwell.edu (M.J.B.); brochels@northwell.edu (B.R.); 2 Department of Obstetrics and Gynecology, North Shore University Hospital, Manhasset, NY 11030, USA; 3 Department of Obstetrics and Gynecology, Long Island Jewish Medical Center, New Hyde Park, NY 11004, USA; 4 Zucker School of Medicine at Hofstra/Northwell, Hempstead, NY 11549, USA; 5 Northwell Health Laboratories, Lake Success, NY 11042, USA; 6 Department of Obstetrics and Gynecology, South Shore University Hospital, Bay Shore, NY 11706, USA

**Keywords:** periviability, counseling, neonatology, resuscitation, intervention, comfort care, termination, social vulnerability index, SVI, maternal factors

## Abstract

**Background/Objectives**: According to the American Academy of Pediatrics and the American College of Obstetricians and Gynecologists, pregnant people facing periviable delivery should be counseled on expected neonatal outcomes and available pregnancy options. The objective of this study is to evaluate if rates of neonatology consultation and pregnancy option counseling for those facing periviable delivery differ based on social vulnerability factors or Social Vulnerability Index (SVI). **Methods**: This was a retrospective cohort study of patients who delivered at 22 0/7 weeks to 25 6/7 weeks of gestation at two academic medical centers with level III or IV neonatal intensive care units from 2019 to 2022. We analyzed the association between individual-level social vulnerability factors and census-tract-level SVI (released by the Center for Disease Control) and the rates of neonatology consultation and pregnancy option counseling. **Results**: In 138 periviable deliveries, 70.3% had a neonatology consultation, 92.0% were offered expectant management, 88.4% were offered neonatal full intervention, 41.3% were offered neonatal comfort care, and 44.9% were offered pregnancy termination. The rate at which neonatology consultations were completed and each pregnancy option was addressed did not differ by individual-level social vulnerability factors including race/ethnicity, government insurance, marital status, English proficiency, and parity, or by SVI. **Conclusions**: While these findings suggest that individual-level social vulnerability factors and SVI do not influence periviability counseling, we did identify gaps in comprehensive periviability counseling. Focus should be placed on increasing the rate of neonatology consultation and discussing the options of neonatal comfort care and pregnancy termination.

## 1. Introduction

Periviability describes the time period during which the fetus may survive outside the uterus with life-sustaining interventions and is defined as 20 0/7 weeks to 25 6/7 weeks of gestation [1,2]. Birth during this time period is associated with significant morbidity, long-term neurodevelopmental impairment, and mortality. Survival to discharge in this period ranges from 6 to 17% at 22 weeks, 26 to 48% at 23 weeks, 55 to 60% at 24 weeks, and 72 to 75% at 25 weeks. Some of the variation in these ranges is explained by differences in the level of neonatal care at the delivering institution and in hospital practices regarding the initiation of active treatment [3,4,5]. Periviable delivery may also be associated with increased pregnant person morbidity including infection, blood transfusion, and unplanned operative procedures including hysterectomy [6,7]. Pregnancies requiring delivery in the periviable period are challenging clinical scenarios for patients, patients’ families, clinicians, and staff. 

In the setting of anticipated or inevitable periviable birth, pregnant people should be counseled on the possible neonatal outcomes, interventions to help improve outcomes, and available options including neonatal full intervention, neonatal comfort care, and pregnancy termination [8,9]. This counseling should be sensitive to patient factors such as socioeconomic status, primary language, religion, and cultural and ethnic background [8]. Counseling should be provided by a multidisciplinary team that includes obstetrician gynecologists; maternal–fetal medicine specialists, if available; and neonatologists, who often provide complementary information [9,10]. Families and healthcare providers face complex, emotionally and ethically challenging decisions in the setting of periviable birth. Periviability counseling must merge medical information on neonatal outcomes, obstetric interventions, and pregnancy options with the patient’s and family’s values and preferences to optimally support their decision making. In ongoing pregnancies, follow-up counseling is recommended to reassess the management plan as the clinical situation evolves and the gestational age increases [9]. 

Individual-level social vulnerability factors including income, education level, marital status, housing insecurity, and race/ethnicity are risk factors for preterm birth [11]. Social vulnerability composites from the individual to county level have additionally been associated with increased risk of preterm birth including extremely preterm birth [12,13,14]. Among these, the Social Vulnerability Index (SVI), developed by the Center for Disease Control, characterizes the overall social vulnerability of each census tract [15]. Social vulnerability factors such as age, socioeconomic status, race/ethnicity, insurance status, education level, marital status, parity, and pregnancy intendedness may influence counseling for periviable pregnancies based on survey-based and simulation-based studies, but to the best of our knowledge clinical studies are not available [16,17,18]. Racial and ethnic differences exist in the utilization of periviable resuscitation; most notably, periviable neonates born to Black and Hispanic people are more likely to be resuscitated than those born to white people [19,20,21,22]. Black race, Hispanic ethnicity, and residential segregation are associated with care in lower-quality neonatal intensive care units and higher neonatal morbidity and mortality for very preterm infants [23,24,25,26]. It is unreported whether social vulnerability composites such as SVI influence periviability counseling; utilization of periviable resuscitation, comfort care, or termination; and rates of neonatal morbidity and mortality.

The objective of this study was to evaluate if periviability counseling differs with individual social vulnerability factors or SVI. Our primary objective was to assess whether neonatology consultation and pregnancy option counseling differed based on social vulnerability. 

## 2. Materials and Methods

This was a retrospective cohort study of patients who delivered at 22 0/7 weeks to 25 6/7 weeks of gestation at two academic medical centers between 1 January 2019, and 31 December 2022. The two medical centers are part of a diverse hospital system in the New York metropolitan area. One study site has a level III neonatal intensive care unit, and the other study site has a level IV neonatal intensive care unit. Patients were excluded if they were less than 18 years old or had a multifetal gestation, fetal anomaly with poor prognosis, or fetal demise. This study received institutional review board approval prior to data collection. 

Data were obtained from the electronic health record system (Sunrise Clinical Manager, Chicago, IL, USA). Patient demographic factors included age, parity, pre-pregnancy body mass index (BMI), race/ethnicity, insurance status, marital status, English proficiency, gestational age on admission, and delivery details including administration of medical interventions to improve neonatal outcomes, gestational age at delivery, indication for delivery, fetal presentation, mode of delivery, neonatal sex, and birth weight. 

Each patient’s address of residence was used to identify their census tract and then linked to SVI scores released by the Center for Disease Control and Agency for Toxic Substances and Disease Registry [15]. No patients were missing SVI data. The SVI score incorporates 16 United States census variables that fall into four major themes: socioeconomic status (income below 150% poverty level, unemployment, education, health insurance), household characteristics (children, senior, disability, single-parent, English proficiency), racial and ethnic minority status, and housing type and transportation (multi-unit structures, mobile homes, crowding, group quarters, no vehicle). Each census tract receives an overall score, as well as a score for each of the four themes, that is between 0 and 1. The higher the SVI score, the higher the social vulnerability of the community. This study reports SVI scores in quartiles (quartile 1 = low vulnerability; quartile 2 = moderately low vulnerability; quartile 3 = moderately high vulnerability; and quartile 4 = high vulnerability). 

**Figure 1:**
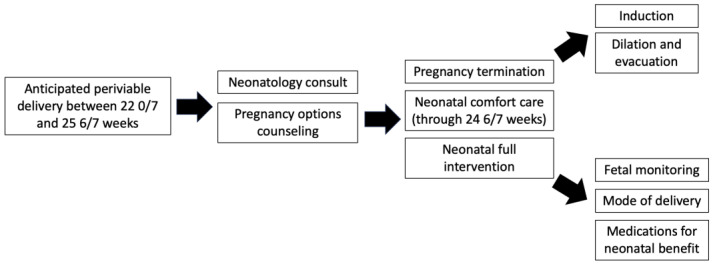
Overview of periviability counseling.

Documentation in the electronic health record of periviability counseling by obstetrician gynecologists, maternal–fetal medicine specialists, and neonatologists was reviewed by two researchers (GP, LC) for all eligible patients. To ensure inter-rater reliability, we utilized a standardized data collection tool, each researcher reviewed ten charts in common with 95% agreement, and we reviewed questions together. Figure 1 illustrates a comprehensive model for periviability counseling and serves as a model for electronic health record review. Documentation was evaluated for counseling on options including expectant management, neonatal full intervention, neonatal comfort care, fetal monitoring, mode of delivery, pregnancy termination, methods of pregnancy termination, and neonatology consultation. 

Primary outcomes were completion of neonatology consultation and provision of options for neonatal full intervention, neonatal comfort care, and pregnancy termination. New York State allows neonatal comfort care through 24 6/7 weeks of gestation and pregnancy termination through 25 6/7 weeks of gestation (and at later gestational ages in specific circumstances). Secondary outcomes were discussion of fetal monitoring, discussion of mode of delivery, and options for methods of pregnancy termination. Descriptive statistics were used to characterize the data. Categorical variables were expressed as frequency and percentage and continuous variables were expressed as median and interquartile range. The chi-square test or Fisher’s exact test were used to examine associations between the categorical variables. Analysis of variance (ANOVA), the Wilcoxon signed-rank test, and the Kruskal–Wallis test were used to examine associations between continuous variables. Given the multiple comparisons, a Bonferroni correction was applied to determine the threshold for significance. All statistical analyses were performed using SAS version 9.4M6 (Cary, NC, USA). 

## 3. Results

**Figure 2:**
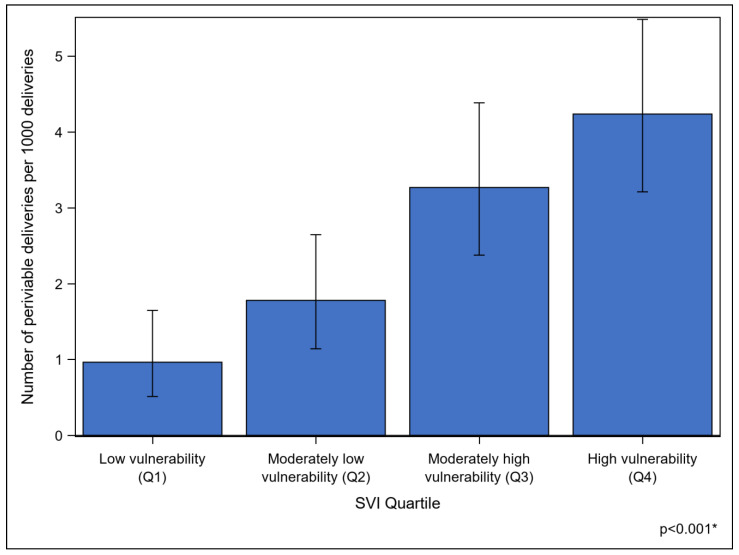
Rate of periviable delivery by SVI quartile from 2019 to 2022. * *p* value determined by chi-square test.

From 2019 to 2022, there were a total of 53,908 deliveries across the two medical centers, 138 of which were periviable deliveries between 22 0/7 weeks and 25 6/7 weeks. The overall periviable delivery rate was 2.5 per 1000 deliveries. The periviable delivery rate was four times higher among high-vulnerability individuals (4.2 per 1000 deliveries) than among low-vulnerability individuals (1 per 1000 deliveries) (Figure 2).

The study cohort consisted of 138 periviable deliveries. Baseline demographics of the patients included are displayed in Table 1. Common patient characteristics included nulliparity (58.0%), non-Hispanic Black race/ethnicity (41.3%), government insurance (44.9%), unmarried status (54.3%), and English proficiency (92.0%). The median overall composite SVI score was 0.65 (0.44, 0.86) with a range of 0.03 to 1.00 and 73.2% of patients were in the moderately high- or high-vulnerability category. There was a direct relationship between non-Hispanic Black race/ethnicity and SVI quartile, but otherwise there were no additional notable differences in any of the individual-level social vulnerability factors and SVI (Supplemental Table S1). The median gestational age on admission was 23 5/7 weeks with a range of 21 6/7 weeks to 25 3/7 weeks and the median gestational age at delivery was 24 2/7 weeks with a range of 22 0/7 weeks to 25 6/7 weeks. Finally, the proportion of female neonates increased with increasing SVI. 

**Figure 3:**
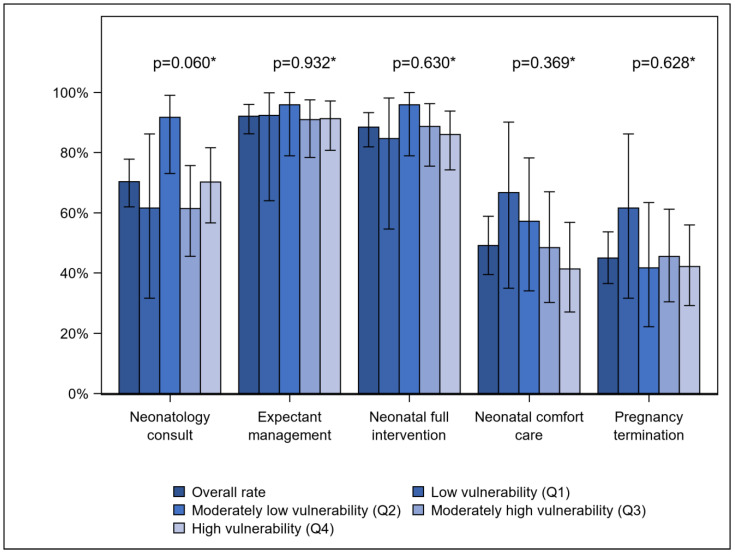
Major components of periviability counseling by SVI quartile. * *p* values determined by chi-square test or Fisher’s exact test; *p* values should be compared to the Bonferroni-corrected critical value of 0.05/5 = 0.010.

Across all patients, 70.3% had a neonatology consultation, 92.0% were offered expectant management, 88.4% were offered neonatal full intervention, 41.3% were offered neonatal comfort care, and 44.9% were offered pregnancy termination. The rate at which neonatology consultations were completed and each of these options were discussed as part of periviability counseling did not differ by SVI quartile (Figure 3) or by individual-level social vulnerability factors including race/ethnicity, government insurance, marital status, nulliparity, and English proficiency (Table 2). The median SVI of those who received neonatology consultation or were offered expectant management, neonatal full intervention, neonatal comfort care, or pregnancy termination did not differ from those who missed any of these components of periviability counseling (Table 3). 

Among those with whom neonatal full intervention was addressed as an option, fetal monitoring was discussed with 33.6% of patients and mode of delivery was discussed with 77.0%. The rate at which fetal monitoring and mode of delivery were addressed did not differ by SVI (Table 4). Additionally, among those who were offered neonatal full intervention, the rates of antenatal corticosteroid, magnesium, and group B strep prophylaxis administration were 77.0%, 79.5%, and 60.7%, respectively, and did not differ by SVI quartile. Among those with whom pregnancy termination was addressed as an option, method of termination was explored with 59.7%. In this group, 25.8% was offered induction termination and dilation and evacuation, 32.3% was just offered induction termination, and 1.6% was just offered dilation and evacuation. The rate at which methods of termination were discussed did not differ by SVI (Table 4). 

## 4. Discussion

Our study suggests that individual-level social vulnerability factors and census-tract-level SVI are not associated with differences in the rate of neonatology consultation or pregnancy options presented during periviability counseling. We identified gaps in periviability counseling given that almost 30% of patients did not have a neonatology consultation and greater than 50% of patients were not counseled on the options for neonatal comfort care or pregnancy termination. 

Our findings demonstrate that more socially vulnerable patients have increased rates of periviable birth compared to less socially vulnerable patients. The existing literature similarly demonstrates demographic differences in preterm birth rates, with Black women at increased risk of periviable birth and women of lower socioeconomic status at increased risk for preterm birth compared to the general patient population [11,27,28]. However, the degree of preterm birth, or the observed gestational age, did not appear to differ based on socioeconomic factors in another study [29]. The reasons for these socioeconomic discrepancies are unknown, with postulated etiologies that include maternal stress, differences in healthcare access, and less available social support [28].

Data evaluating the association between social vulnerability factors and periviability counseling are limited. Previous studies have suggested that social vulnerability factors may impact periviability counseling for pregnant people [17,18,30]. Two nationwide surveys asked neonatologists how likely they were to recommend full resuscitation versus comfort care in response to clinical vignettes of periviable pregnancies that varied in specific social vulnerability factors [17,30]. They found that counseling was influenced by parity, pregnancy intendedness, and socioeconomic status, but not by race, education, or age. Additionally, a simulation-based study including both obstetricians and neonatologists explored periviability counseling with a focus on resuscitation, mode of delivery, and steroids for standardized patients of varied race and insurance status, both of which were found to impact management decision making [18]. 

To the best of our knowledge, this study is the first to explore the association of individual-level social vulnerability factors and community-level SVI and periviability counseling in the clinical setting. We did not find an association between race/ethnicity, government insurance, marital status, nulliparity, and English proficiency or between SVI and periviability counseling. Our results were unanticipated and contrast the above studies that suggest possible implicit provider bias in management of patients at risk for periviable birth [17,18,30]. The lack of statistically significant differences could be due to the small sample size and a skew in our population towards higher social vulnerability. Another possible reason for our findings is the presence of a standardized approach for the evaluation and management of periviable pregnant patients at the study institutions. At both institutions, it is standard to consult both maternal–fetal medicine and neonatology for counseling of a patient at risk for periviable birth. It is also possible that workplace interventions such as implicit bias training have contributed to improved consistency in periviability counseling. 

The American College of Obstetricians and Gynecologists and the Society for Maternal-Fetal Medicine recommend that counseling in periviable gestations should be provided by a multidisciplinary team that includes obstetrician gynecologists, maternal–fetal medicine specialists, and neonatologists and should address short- and long-term neonatal outcomes and available pregnancy options [9]. According to this study and one previous retrospective cohort study that we could identify, there remains variability in neonatology counseling in those at risk of periviable delivery. In the previous study of 498 pregnant people delivering between 22 and 24 weeks of gestation at six United States medical centers, 62% had a documented neonatology consultation with the rate increasing from 40% at 22 weeks of gestation to 72% at 24 weeks of gestation [31]. In our study, the rate of neonatology consultation was slightly higher at 70%. The rate increased from 40% at 22 weeks of gestation to 84% at 24 weeks of gestation. Short admission-to-delivery time intervals only partially explain these neonatology consultation gaps, accounting for 40% of those lacking neonatology consultation in our study. One possible explanation for the remaining gap in completion of neonatology consultations includes inconsistent classification of neonates prior to 23 weeks of gestation as previable. Approximately 28% of neonates born prior to 23 weeks were considered previable by the obstetrics team and therefore neonatology consultation was not offered. Additional explanations for the gap may also include patients declining consultation or choosing pregnancy termination, the obstetrics team not requesting a consultation, and the neonatology team not completing a requested consultation. Consistently ensuring timely neonatology consultation starting at 22 weeks of gestation is vital for comprehensive periviability counseling and informed patient decision making. 

There also remains a gap in comprehensive pregnancy option counseling including the choices of neonatal full intervention, neonatal comfort care, and pregnancy termination for those at risk of periviable delivery. Most notably in our study, only 41.3% of pregnant people were offered neonatal comfort care and 44.9% of people were offered pregnancy termination. Possible explanations for gaps in comprehensive pregnancy option counseling are provider and/or institutional discomfort with comfort care and pregnancy termination, particularly with advancing gestational age; provider discretion based on interpretation of patient preferences; and incomplete documentation. None of the previous studies exploring social vulnerability factors and periviability counseling addressed the option of pregnancy termination [17,18,30]. We additionally recommend a focus on providing full-option counseling, including neonatal full intervention, neonatal comfort care, and pregnancy termination, when clinically applicable based on gestational age for those at risk of periviable delivery. 

Our study has several strengths. First, the electronic health record system is the same across both hospitals, which allows for uniformity in data collection. Next, the two hospitals care for a diverse patient population that resides in both urban and suburban communities. Both neonatal intensive care units can provide comprehensive care for neonates born at all gestational ages. Additionally, New York State allows neonatal comfort care through 24 6/7 weeks and pregnancy termination through 25 6/7 weeks (and beyond in specific circumstances), which enables expansive option counseling given the ability to provide neonatal full intervention, neonatal comfort care, and pregnancy termination in the periviable period. We also looked at both individual-level social vulnerability factors and community-level SVI. Finally, SVI data are publicly available and can be easily applied in other regions across the United States. 

The biggest limitation of this study is its retrospective nature. We relied on medical documentation to characterize the periviability counseling, which may not fully capture everything stated at the time of the actual counseling. At the medical centers in this study, neonatology consult notes are templated while obstetrics and gynecology and maternal–fetal medicine notes are free-text. There is also the possibility of inter-researcher variability in characterizing consultation findings during reviews of electronic health records. Residents, fellows, advanced care practitioners, and attendings can all be involved in periviability counseling and may differ in approach and content. An additional limitation is the small sample size due to the rarity of periviable delivery. Larger multicenter studies may be needed to reach firm conclusions, but may also be limited by state differences in legal gestational age limits for neonatal comfort care and pregnancy termination and local differences in their approach to resuscitation of periviable neonates. Finally, due to the rarity of periviable birth, each census tract was represented by only one or two patients, and as a result a multilevel model evaluating neighborhood effects could not be directly evaluated.

## 5. Conclusions

While high-vulnerability individuals are disproportionately impacted by periviable birth, individual-level social vulnerability factors and census-tract-level SVI do not influence periviability counseling. Neonatology consultation was completed and options for neonatal full intervention, neonatal comfort care, and pregnancy termination were provided equally regardless of social vulnerability.

However, we identified gaps in pregnancy option counseling across this group of pregnant people with periviable deliveries. To best support patients facing periviable birth in their decision making, it is imperative that they have a complete understanding of neonatal morbidity and mortality through neonatology consultation and be presented with full pregnancy options including neonatal comfort care and pregnancy termination, all of which were variably incorporated into counseling in this study. Further research is needed to identify effective interventions to promote unbiased, comprehensive periviability counseling.

## Figures and Tables

**Table 1:** Patient characteristics.

Characteristics	Patients n = 138
Maternal age (years)	32.3 (28.1, 36.8)
Nulliparous	80 (58.0)
Pre-pregnancy BMI	29.2 (24.4, 32.8)
Race/Ethnicity	
Non-Hispanic White	21 (15.2)
Non-Hispanic Black	57 (41.3)
Other/Unknown	60 (43.5)
Government insurance	62 (44.9)
Married	63 (45.7)
English proficiency	127 (92.0)
Year of delivery	
2019	33 (23.9)
2020	36 (26.1)
2021	33 (23.9)
2022	36 (26.1)
Gestational age on admission (weeks)	23.8 (22.9, 24.9)
Gestational age at delivery (weeks)	24.3 (23.2, 25.1)
Spontaneous preterm delivery	114 (82.6)
Cephalic presenting fetus	67 (48.9) [n = 137]
Female neonate	63 (46.0) [n = 137]
Birth weight (grams)	655.0 (530.0, 780.0) [n = 133]

Categorical variables listed as number (%). Continuous variables listed as median (interquartile range).

**Table 2:** Major components of periviability counseling by individual-level social vulnerability factors.

Characteristic	Neonatology Consult	Expectant Management	Neonatal Full Intervention	Neonatal Comfort Care	Pregnancy Termination
Race/Ethnicity					
Non-Hispanic White [n = 21]	10 (47.6)	18 (85.7)	19 (90.5)	9 (60.0) [n = 15]	7 (33.3)
Non-Hispanic Black [n = 57]	39 (68.4)	52 (91.2)	50 (87.7)	17 (37.8) [n = 45]	22 (38.6)
Other/Unknown [n = 60]	48 (80.0)	57 (95.0)	53 (88.3)	28 (56.0) [n = 50]	33 (55.0)
*p* value *	0.019	0.334	0.944	0.137	0.104
Government insurance [n = 62]	42 (67.7)	60 (96.8)	52 (83.9)	27 (52.9) [n = 51]	29 (46.8)
*p* value *	0.554	0.111	0.133	0.453	0.694
Married [n = 63]	49 (77.8)	61 (96.8)	58 (92.1)	27 (52.9) [n = 51]	28 (44.4)
*p* value *	0.078	0.057	0.219	0.453	0.917
Nulliparous [n = 80]	59 (73.8)	73 (91.3)	71 (88.8)	33 (51.6) [n = 64]	38 (47.5)
*p* value *	0.296	0.761	0.882	0.541	0.476
English proficiency [n = 127]	89 (70.1)	116 (91.3)	112 (88.2)	51 (50.5) [n = 101]	57 (44.9)
*p* value *	1.000	0.600	1.000	0.490	1.000

Number (%). * *p* values determined by chi-square test or Fisher’s exact test; *p* values should be compared to the Bonferroni-corrected critical value of 0.05/25 = 0.002.

**Table 3:** Comparison of median SVI between those who received each major component of periviability counseling and those who did not.

Components of Periviability Counseling	Median SVI Among Those Whose Counseling Included Components	Median SVI Among Those Whose Counseling Excluded Components	*p* Value *
Neonatology consult	0.65 (0.44, 0.85)	0.65 (0.53, 0.86)	0.643
Expectant management	0.65 (0.44, 0.86)	0.62 (0.53, 0.81)	0.972
Neonatal full intervention	0.65 (0.44, 0.83)	0.73 (0.545, 0.93)	0.440
Neonatal comfort care	0.57 (0.26, 0.81)	0.70 (0.52, 0.92)	0.081
Pregnancy termination	0.62 (0.42, 0.81)	0.67 (0.47, 0.87)	0.336

Median (interquartile range). * *p* values determined by Wilcoxon signed-rank test; *p* values should be compared to the Bonferroni-corrected critical value of 0.05/5 = 0.010.

**Table 4:** Follow-up components of periviability counseling by SVI quartile.

Components of Periviability Counseling	All n = 138	Low Vulnerability (Q1) n = 13	Moderately Low Vulnerability (Q2) n = 24	Moderately High Vulnerability (Q3) n = 44	High Vulnerability (Q4) n = 57	*p* Value *
Discussed fetal monitoring	41 (33.6) [n = 122]	3 (27.3) [n = 11]	6 (26.1) [n = 23]	15 (38.5) [n = 39]	17 (34.7) [n = 49]	0.749
Discussed mode of delivery	94 (77.0) [n = 122]	6 (54.5) [n = 11]	21 (91.3) [n = 23]	27 (69.2) [n = 39]	40 (81.6) [n = 49]	0.052
Discussed pregnancy termination via induction	36 (58.1) (n = 62)	5 (62.5) [n = 8]	5 (50.0) [n = 10]	11 (55.0) [n = 20]	15 (62.5) [n = 24]	0.915
Discussed pregnancy termination via dilation and evacuation	17 (27.4) (n = 62)	4 (50.0) [n = 8]	1 (10.0) [n = 10]	4 (20.0) [n = 20]	8 (33.3) [n = 24]	0.230
Betamethasone administered	94 (77.0) [n = 122]	5 (45.5) [n = 11]	20 (87.0) [n = 23]	28 (71.8) [n = 39]	41 (83.7) [n = 49]	0.025
Magnesium administered	97 (79.5) [n = 122]	6 (54.5) [n = 11]	20 (87.0) [n = 23]	29 (74.4) [n = 39]	42 (85.7) [n = 49]	0.092
Group B strep prophylaxis administered	74 (60.7) [n = 122]	6 (54.5) [n = 11]	14 (60.9) [n = 23]	20 (51.3) [n = 39]	34 (69.4) [n = 49]	0.366
Delivered via cesarean section	69 (50.4)	5 (38.5)	15 (62.5)	21 (48.8) [n = 43]	28 (49.1)	0.527

Number (%). * *p* values determined by chi-square test or Fisher’s exact test; *p* values should be compared to the Bonferroni-corrected critical value of 0.05/8 = 0.006.

## Data Availability

The raw data supporting the conclusions of this article will be made available by the authors on request.

## Supplementary Materials

The following supporting information can be downloaded at https://www.mdpi.com/article/10.3390/jcm14020466/s1: Table S1: Patient characteristics by SVI quartile.
