# Supplementary material for: Social Vulnerability and Pregnancy Option Counseling in the Setting of Periviable Delivery
Source: J Clin Med. 2025 Jan 13;14(2):466. doi: 10.3390/jcm14020466 (PMC11766277; doi:10.3390/jcm14020466)
Supplement: Supplementary file 1 [file jcm-14-00466-s001.zip › jcm-3361822-supplementary.pdf]

Supplemental Table S1. Patient characteristics by SVI quartile.

Characteristics	Low vulnerability (Q1) n = 13	Moderately low vulnerability (Q2) n = 24	Moderately high vulnerability (Q3) n = 44	High vulnerability (Q4) n = 57	p value*
Maternal age (years)	33.2 (28.6,37.9)	34.2 (31.2,39.7)	31.0 (27.6,35.0)	32.0 (29.8,36.0)	0.071
Nulliparous	5 (38.5)	12 (50.0)	26 (59.1)	37 (64.9)	0.283
Pre-pregnancy BMI	24.0 (21.9,29.0)	27.4 (24.6,34.8)	29.7 (24.0,31.1)	30.0 (25.4,34.5)	0.103
Race/Ethnicity					
Non-Hispanic White	7 (53.9)	5 (20.8)	6 (13.6)	3 (5.3)	0.001
Non-Hispanic Black	1 (7.7)	8 (33.3)	17 (38.6)	31 (54.4)	
Other/Unknown	5 (38.5)	11 (45.8)	21 (47.7)	23 (40.4)	
Government insurance	2 (15.4)	9 (37.5)	20 (45.5)	31 (54.4)	0.066
Married	8 (61.5)	15 (62.5)	18 (40.9)	22 (38.6)	0.132
English proficiency	13 (100.0)	21 (87.5)	39 (88.6)	54 (94.7)	0.442
Year of delivery					
2019	1 (7.7)	7 (29.2)	13 (29.6)	12 (21.1)	0.417
2020	3 (23.1)	8 (33.3)	8 (18.2)	17 (29.8)	
2021	5 (38.5)	6 (25.0)	8 (18.2)	14 (24.6)	
2022	4 (30.8)	3 (12.5)	15 (34.1)	14 (24.6)	
Gestational age on admission (weeks)	23.0 (22.6,24.3)	23.6 (23.1,24.2)	24.2 (23.1,25.1)	23.6 (22.9,24.7)	0.264
Gestational age at delivery (weeks)	24.0 (22.4,24.3)	24.4 (23.7,25.0)	24.4 (23.3,25.2)	24.2 (23.2,25.2)	0.181
Spontaneous preterm birth	12 (92.3)	21 (87.5)	33 (75.0)	48 (84.2)	0.455
Cephalic-presenting fetus	8 (61.5)	10 (41.7)	19 (44.2) [n = 43]	30 (52.6)	0.566
Female neonate	4 (30.8)	5 (20.8)	22 (50.0)	32 (57.1) [n = 56]	0.015
Birth weight (grams)	640.0 (515.0,730.0)	692.5 (580.0,815.0)	645.0 (510.0,770.0) [n = 41]	630.0 (532.0,780.0) [n = 55]	0.467

Categorical variables listed as number (%).
For categorical variables, p values were determined by chi-square test or Fisher's exact test.
Continuous variables are listed as median (interquartile range).
For continuous variables, p values were determined by ANOVA or Kruskal–Wallis test.
*p <0.05 is considered statistically significant.
